# YAP/TAZ Activation as a Target for Treating Metastatic Cancer

**DOI:** 10.3390/cancers10040115

**Published:** 2018-04-10

**Authors:** Janine S. A. Warren, Yuxuan Xiao, John M. Lamar

**Affiliations:** Department of Molecular and Cellular Physiology, Albany Medical College, Albany, NY 12208, USA; warrenj1@amc.edu (J.S.A.W.); xiaoy@amc.edu (Y.X.)

**Keywords:** YAP, TAZ, metastasis, hippo pathway

## Abstract

Yes-Associated Protein (YAP) and Transcriptional Co-activator with PDZ-binding Motif (TAZ) have both emerged as important drivers of cancer progression and metastasis. YAP and TAZ are often upregulated or nuclear localized in aggressive human cancers. There is abundant experimental evidence demonstrating that YAP or TAZ activation promotes cancer formation, tumor progression, and metastasis. In this review we summarize the evidence linking YAP/TAZ activation to metastasis, and discuss the roles of YAP and TAZ during each step of the metastatic cascade. Collectively, this evidence strongly suggests that inappropriate YAP or TAZ activity plays a causal role in cancer, and that targeting aberrant YAP/TAZ activation is a promising strategy for the treatment of metastatic disease. To this end, we also discuss several potential strategies for inhibiting YAP/TAZ activation in cancer and the challenges each strategy poses.

## 1. Introduction

Yes-associated protein (YAP) and transcriptional co-activator with PDZ-binding motif (TAZ) are transcriptional co-activators that are negatively regulated by the Hippo pathway. YAP was identified and cloned in 1994 as a protein that associates with the Src family non-receptor tyrosine kinase Yes [1]. TAZ was identified and described six years later as a protein that binds 14-3-3 [2]. Since their discovery, YAP and TAZ have emerged as critical regulators of early embryonic development [3,4,5,6], as well as the development and growth of several tissue types [3,5,6]. YAP and TAZ also have important roles in adult organs, particularly during tissue repair and regeneration [5,6,7]. YAP and TAZ mediate these processes by driving the transcription of genes that promote cell proliferation, cell survival, and stem cell maintenance. In contrast, repression of YAP and TAZ by the Hippo pathway restrains cell proliferation and promotes differentiation to maintain proper organ size. Like many growth regulatory pathways, dysregulation of the Hippo-YAP/TAZ pathway has severe consequences. Inappropriately low YAP/TAZ activity can lead to developmental defects, tissue atrophy, and defective tissue repair, whereas aberrantly high YAP/TAZ activity promotes tissue overgrowth and tumor formation. Here, we discuss how abnormal YAP/TAZ activity promotes cancer formation, progression, and metastasis, and how YAP/TAZ and their upstream regulators or their downstream target genes are potential therapeutic targets for preventing and treating metastatic cancer. 

## 2. YAP and TAZ and Their Regulation

The core Hippo pathway proteins and their role in organ size regulation was first elucidated in *Drosophila* using genetic screens. Loss of function mutations in any one of the genes that encode, Warts, Salvador, mob-as-tumor suppressor, or Hippo (Hpo) each resulted in significant tissue overgrowth [8,9,10,11,12,13,14,15,16,17]. A subsequent study identified Yorkie (Yki), the *Drosophila* ortholog of YAP and TAZ, as the downstream effector of the *Drosophila* Hippo pathway that promotes tissue growth [18]. The Hippo pathway and its regulation of Yorkie are largely conserved in vertebrates where YAP and TAZ are phosphorylated and repressed by the Hippo pathway core kinase cascade [19]. Activated Mammalian Sterile 20-like Kinase 1 (MST1) or Mammalian Sterile 20-like Kinase 2 (MST2), the mammalian orthologs of Hpo, bind and phosphorylate their scaffold protein Salvador Homolog 1 (SAV1) [9,20]. Together, the MST/SAV1 complex then phosphorylates and activates the downstream kinases Large Tumor Suppressor Homolog 1 (LATS1) and Large Tumor Suppressor Homolog 2 (LATS2), as well as their scaffold proteins MOB Kinase Activator 1A and 1B (MOB1A and MOB1B) [8,21]. The active LATS/MOB complex can then bind and phosphorylate YAP and TAZ to prevent nuclear translocation or promote protein degradation. Although LATS can phosphorylate YAP on 5 distinct serine residues (4 serines in TAZ), most LATS-mediated repression of YAP and TAZ is mediated by two serines. Phosphorylation of YAP on serine 127 or TAZ on serine 89 promotes 14-3-3 binding and cytoplasmic sequestration [2,22,23]. Phosphorylation of serine 381 on YAP or serine 311 on TAZ promotes subsequent phosphorylation by Casein Kinase I δ/ε and leads to the recruitment of the E3 ubiquitin ligase SCF(β-TRCP), which leads to ubiquitination and proteasomal degradation [24,25]. TAZ protein stability can be further regulated by GSK-mediated phosphorylation in an N-terminal phosphodegron that also recruits SCF(β-TRCP) [26]. Non-phosphorylated YAP and TAZ can enter the nucleus to promote the expression of target genes; however, both YAP and TAZ lack DNA binding domains and must therefore interact with other transcription factors to drive transcription. Although YAP and TAZ are known to interact with numerous transcription factors [27], the TEA family members (TEADs 1–4) appear to mediate many YAP/TAZ-dependent processes [27,28,29,30].

There is a long and growing list of proteins and pathways that collectively regulate YAP and TAZ in response to a diverse set of extracellular cues (for reviews see [3,31,32,33,34,35,36]). This includes several proteins that are considered important upstream components of the Hippo pathway in flies and/or vertebrates such as Tao kinases, FAT atypical cadherins 1–4 (FAT 1–4), WW and C2 Domain Containing 1 (WWC1) and 2 (WWC2), FE1-4RM Domain-containing Proteins 1 and 6 (FRMD1 and FRMD6), Dachshund Homologs 1 and 2, Dachsous, Neurofibromin 2 (NF2)/Merlin, and Zonula Occludens 1 and 2. Adherens junction proteins such as E-cadherin, α-catenin, and β-catenin also influence the Hippo pathway. Apical-basal polarity proteins including Crumbs Homologs 1–3, Lethal Giant Larvae 1 and 2, atypical Protein Kinases Cλ and Cζ, and Scribble can regulate the Hippo pathway as well. In addition, several other important cellular pathways can influence YAP and TAZ activity in response to mechanical cues, integrin-extracellular matrix (ECM) adhesion, altered metablism, G protein-coupled receptor (GPCR) signaling, and growth factor signaling. Most of these pathways regulate YAP/TAZ activity by regulating the Hippo Pathway, but there are also several examples of proteins that regulate YAP and TAZ independent of the Hippo pathway. For a more thorough review of the studies identifying these and other regulators of the Hippo pathway and YAP and TAZ themselves, see the above reviews. Below we highlight examples of how dysregulation of a few of these YAP/TAZ regulatory pathways can promote cancer development and metastasis. 

## 3. YAP/TAZ-TEAD Drives Cancer Formation, Tumor Growth, and Metastasis

Over the past decade, YAP and TAZ have emerged as important drivers of cancer development, tumor growth, and metastasis. Numerous papers, using in vitro studies with human and mouse cancer cell lines or mouse models of cancer, have established roles for inappropriate YAP or TAZ activity in virtually every cancer-associated process. This is accompanied by countless studies that analyze human cancer samples for correlations between YAP/TAZ expression and patient prognosis. Much of the work to date has focused on cancer development and tumor growth, and several recent reviews discuss these studies in detail [4,5,29,32,37,38,39,40]. Though we briefly summarize this work, our discussion focuses more on the studies that implicate YAP and TAZ in cancer metastasis. 

### 3.1. YAP/TAZ-TEAD Activation Promotes Tumor Formation and Growth

Studies that analyzed YAP and TAZ mRNA, protein expression, and/or nuclear localization using immunohistochemistry or gene expression analysis have overwhelmingly found that expression or activity of YAP or TAZ is increased in many human cancers compared to corresponding normal tissue (reviewed in [5,40,41]). In addition, several of these same studies have demonstrated that this increased YAP and TAZ expression or activity is strongly correlated with poor prognosis. A meta-analysis of 21 different studies with a combined 2983 patients revealed that YAP is overexpressed and associated with poor outcome and reduced survival in many human cancers [42]. A similar meta-analysis of 15 studies comprised of 2881 patients showed that high TAZ expression is correlated with poor survival [43]. Some of this increased expression and activity can be explained by genetic alterations in the Hippo pathway or by amplifications of YAP and TAZ themselves. Indeed, one of the first studies implicating YAP in cancer identified it as the protein-coding gene in a 350-kilobase amplicon found in a mouse mammary tumor [44], and YAP is also on the syntenic human amplicon (chromosome 11q22) that is amplified in a variety of cancer types [45,46,47,48,49,50,51,52,53,54,55]. TAZ amplifications occur in human cancer as well [56,57]. Furthermore, chromosomal translocations that generate fusion proteins containing TAZ (WWTR1-CAMAT1) or YAP (YAP-TFE3) are known disease-driving events in a vascular sarcoma called epithelioid hemangioendothelioma [58,59,60]. Mutations in several core Hippo pathway proteins including LATS1 [61,62,63,64,65], LATS2 [64,66,67,68,69,70], MST1 or MST2 [71,72,73], SAV1 [13], and MOB1A or MOB1B [74] exist, and evidence suggest that several of these genes can be repressed epigenetically [75,76,77]. Thus, genetic alterations in the Hippo-YAP/TAZ pathway are present in a variety of human cancer types. However, these genetic alterations are not common enough to fully explain the frequency of increased YAP/TAZ expression and nuclear localization, which suggests that other cancer-associated pathways can promote inappropriate YAP/TAZ activity. 

There is a wealth of in vitro evidence showing that YAP and/or TAZ activation promotes cancer cell proliferation, anchorage-independent growth, and cellular transformation in a variety of different cancer cell lines. This is complemented by numerous in vivo studies that show a role for YAP or TAZ in tumor formation and growth. Many of these studies used xenograft model systems with cancer cells that are either overexpressing wild type or LATS-insensitive YAP or TAZ [78,79,80,81,82,83,84,85,86,87,88,89,90,91,92,93], or in which YAP or TAZ have been knocked down [79,84,85,90,94,95,96,97,98,99,100,101]. There are also several studies done in transgenic mice with tissue-specific expression of either wild type or LATS-insensitive YAP or TAZ that show increased tumor formation and enhanced growth [19,84,102,103,104,105]. Expression of Hippo pathway-insensitive Yki or YAP also causes tumor formation in *Drosophila* [99,106]. Knockout of MST1 or MST2 [16,107,108], LATS1 or LATS2 [109], SAV1 [108], MOB [110,111], or NF2 [104,112] also enhances tumor formation and growth and, in many cases, this was YAP/TAZ-dependent. Conversely, tissue-specific knockout of YAP or TAZ inhibits tumor formation [105,113]. Several of the above studies also show that these effects of YAP and TAZ are mediated by TEADs [78,104,114,115,116]. Collectively, these studies clearly demonstrate that inappropriate YAP/TAZ activity is a driver of cancer formation and growth.

### 3.2. YAP/TAZ-TEAD Activation Promotes Metastasis

**Figure 1:**
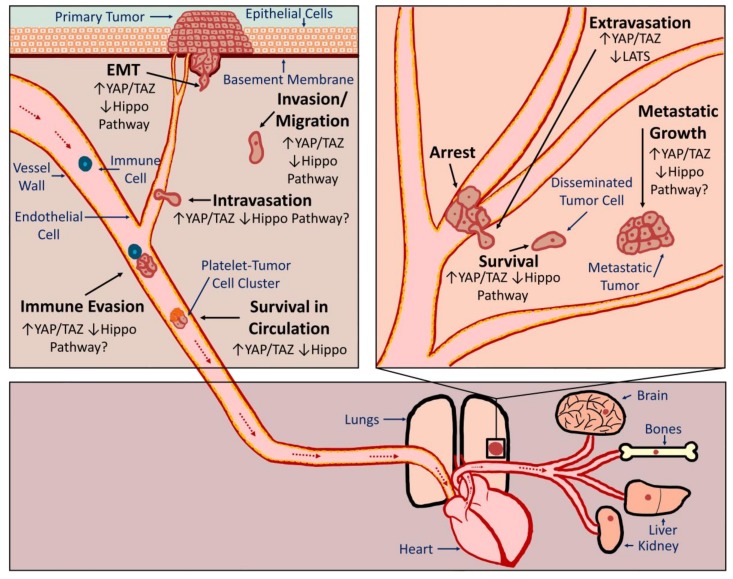
YAP/TAZ activation promotes several steps of the metastatic cascade. Depicted are the critical steps in the metastatic cascade. To spread tumor cancer cells (brown) undergo an EMT and then must invade through basement membranes and the surrounding tissue until they encounter a blood or lymphatic vessel. To intravasate they must then invade between endothelial cells (yellow). While in circulation, tumor cells interact with immune cells (blue) and platelets (orange). To seed distant organs, cells must arrest either by becoming lodged in small capillaries, or through active adhesion to the vessel wall, and then successfully exit the vessel (extravasation). To form a metastatic tumor, the cancer cell must survive and proliferate in a new microenvironment. Whether increased YAP/TAZ activity or decreased Hippo pathway activity promotes each of these steps is indicated. The direction of blood flow is indicated by red dotted arrows.

The vast majority of cancer-associated deaths are the result of metastasis, the spread of cancer cells from the primary tumor to secondary organs. In order to form metastatic tumors, cancer cells must acquire novel abilities that enable them to successfully accomplish a series of steps often referred to as the metastatic cascade (Figure 1). Cancer cells must detach from the primary tumor, invade surrounding tissue, enter a blood or lymphatic vessel (intravasation), evade the immune system, survive in suspension as they circulate, arrest in a distant organ, exit the vessel (extravasation), and then be equipped to survive and grow in a new tissue microenvironment. As discussed in detail below, evidence suggests that inappropriate YAP or TAZ activation can promote metastasis by influencing many of these processes (Figure 1). It should be noted that tumor cells can spread through either hematogenous or lymphogenous mechanisms. Many of the studies discussed below demonstrate a role for YAP or TAZ in metastasis using experimental metastasis models in which tumor cells are injected directly into circulation. This suggests that YAP or TAZ activation can enhance hematogenous metastasis. Although YAP/TAZ activation is correlated with lymph node metastasis, no studies to date have directly investigated whether YAP or TAZ can promote lymphogenous metastasis.

Our 2012 publication was the first evidence that, in addition to promoting tumor growth, YAP activation is sufficient to drive cancer metastasis [78]. Using mutant YAP constructs and multiplex in vivo metastasis assays, we showed that YAP-mediated melanoma and breast cancer metastasis requires YAP-TEAD interaction [78]. We also found that YAP activation promotes transformation, proliferation, migration, invasion, and tumor growth in a TEAD-dependent manner [78]. Another study published at that time also found that activation of YAP, in this case through loss of Leukemia Inhibitory Factor Receptor (LIFR), promoted metastatic colonization of breast cancer cells [117]. Since these initial studies, several others have directly implicated YAP, TAZ, or TEADs in metastasis of numerous cancer types, including melanoma [118], lung cancer [119,120,121], breast cancer [86,122,123,124,125,126,127,128], cholangiocarcinoma [82,129], gastric cancer [130,131,132,133], ovarian cancer [134], colorectal cancer [135,136,137], and oral squamous cell carcinoma [101]. Conversely, LATS1 overexpression reduces gastric cancer metastasis [138]. This experimental data is supported by human patient data showing that YAP or TAZ expression or nuclear localization is increased in metastatic tumors when compared to primary tumors in pancreatic cancer [87], breast cancer [86,139], and prostate cancer [140]. Although most studies suggest that YAP and TAZ expression are associated with poor prognosis and metastasis, some found that YAP expression is inversely correlated with metastasis and prognosis [141,142]. In addition, there are studies that suggest that YAP/TAZ activation can repress metastasis [143,144]. Thus, YAP/TAZ activation drives metastasis in many, but likely not all cancers.

#### 3.2.1. EMT

In order to metastasize, cells must detach from the primary tumor, which often occurs through a process called epithelial to mesenchymal transition (EMT). EMT is typically accompanied by a rearrangement of the cytoskeleton, altered apical-basal cell polarity, and loss of cell-cell adhesion. There are well-described changes in gene expression that are associated with EMT, and several EMT-inducing transcription factors have been identified (reviewed in [145]). Numerous studies have demonstrated roles for YAP and TAZ in EMT (see [31,40,41,146]). Collectively, these studies show that, in cancer cells, increased YAP or TAZ expression or activation disrupts cell-cell junctions, promotes mesenchymal gene expression, and enhances the morphological changes associated with EMT. Conversely, loss of YAP or TAZ, or overexpression of proteins that repress YAP and TAZ, inhibits EMT. Several of the studies discussed in these reviews also show that LATS-insensitive forms of YAP or TAZ can no longer promote EMT if unable to bind TEADs, suggesting that TEADs play a critical role in YAP/TAZ-mediated EMT. Consistently, TEAD knockdown reverses EMT [147]. YAP and TAZ likely drive EMT through a variety of downstream target genes. This includes several EMT-inducing transcription factors such as Forkhead box C2, Snail Family Zinc Finger 1 and 2 (SNAIL, SLUG), Twist-related Protein 1 (Twist), and Zinc Finger E-box-binding Homeobox 1 (ZEB1) [100,139,148,149]. Together, these studies suggest that targeting YAP/TAZ-TEAD in cancer cells could prevent EMT to slow or inhibit metastatic spread.

#### 3.2.2. Migration, Invasion and Intravasation

Following EMT, cancer cells must next invade the surrounding tissue and enter circulation. There is abundant evidence that YAP and TAZ can drive tumor cell migration and invasion (reviewed in [5,34,41,150,151,152]). These studies show that in a variety of cancer cell types, overexpression of wild type or LATS-insensitive YAP or TAZ promotes cell migration and invasion, whereas knockdown of YAP and/or TAZ reduces migration and invasion. As with EMT, YAP/TAZ-mediated migration and invasion is dependent upon TEADs. Importantly, several studies that demonstrate YAP or TAZ-dependent metastasis formation also showed that YAP or TAZ promotes the migration and/or invasion of the cancer cells [82,86,118,120,125,128,129,132,133,135,136,137]. Although the YAP/TAZ target genes responsible for enhanced tumor cell migration and invasion are likely numerous, context-dependent, and cancer-type specific, some have been clearly identified. For example, a recent study found Rho GTPase activating protein 29 (ARHGAP29), a pro-metastatic YAP target gene in gastric cancer, suppresses a RhoA-LIM Domain Kinase (LIMK)-cofilin pathway to drive cell migration [130]. YAP was also found to promote pancreatic cancer cell motility, invasion, and tumorigenesis through LPA Receptor 3 (LPAR3) [87], and TAZ-TEAD-dependent expression of Bone Morphogenic Protein 4 (BMP4) promotes mammary cell migration [153]. YAP/TAZ-TEAD-mediated expression of Receptor of Hyaluronan-Mediated Motility (RHAMM) promotes migration and invasion of mesothelioma and breast cancer cells [154,155], and Zyxin was found to promote breast cancer cell migration and invasion during YAP/TAZ-TEAD2-mediated EMT [127]. Furthermore, TAZ-TEAD mediated repression of ΔNp63 can also promote breast cancer cell migration [156]. Numerous other genes that are regulated by YAP and TAZ have established roles in cell migration and invasion, but the importance of these genes in YAP/TAZ-TEAD-mediated cancer progression has not been tested. Consistent with this experimental data, several studies have demonstrated a strong correlation between YAP or TAZ expression and the invasiveness of human cancers [86,87,140,155,157,158,159,160,161]. 

In order to disseminate to distant sites, cancer cells must intravasate into blood or lymphatic vessels, a process that requires tumor cells to invade through a layer of smooth muscle cells, a basement membrane, and a layer of endothelial cells. Although not thoroughly investigated to date, a few studies have implicated YAP in this process. Loss of LATS1-mediated repression of YAP was found to promote endothelial transmigration of breast cancer cells in vitro and in vivo, whereas YAP knockdown prevented it [126]. Additionally, tumor cell intravasation was increased in a mouse model of uveal melanoma harboring a GNAQ mutation that activates YAP [162]. Neither of these studies determined how YAP activation enhances intravasation, and the role of TAZ in intravasation is unknown. Since tumor cells must migrate through the junctions between endothelial cells in order to intravasate, it seems likely that one way YAP and TAZ enhance intravasation is by promoting cell migration. However, intravasation also requires a loosening of endothelial junctions, which can be enhanced by tumor cells or by stromal cells recruited by tumor cells. Whether any YAP/TAZ target genes influence this process has not yet been directly investigated.

#### 3.2.3. Survival in Circulation and Extravasation

Circulating tumor cells (CTCs) must endure mechanical stress, immune surveillance, and the induction of anoikis caused by loss of cell-ECM adhesion in order to survive. Assaying survival in circulation in vivo is challenging, but studies using surrogate assays suggest that YAP and TAZ can enhance tumor cell survival in circulation. Indeed, while loss of adhesion represses YAP and TAZ to promote anoikis in non-transformed cells, cancer cells with high YAP/TAZ activity are resistant to anoikis [163]. Countless other studies also show that YAP or TAZ activation promotes anchorage-independent growth and/or resistance to anoikis. Furthermore, several studies show that cancer cells in which YAP or TAZ promote anchorage-independent growth have enhanced metastatic [78,118,121,125].

Tumor cells encounter immune cells in the primary tumor as well as during every step of the metastatic cascade, and the ability of a tumor cell to evade or suppress the immune system is critical for tumor progression and metastasis. Programmed Death-Ligand 1 (PD-L1) expression on cancer cells promotes immune suppression by repressing T-cell function [164]. YAP and TAZ both increase PD-L1 expression in several cancer cell types [165,166,167,168,169] and this was shown to be TEAD-dependent [168,169]. YAP activation in cancer cells can also increase the secretion of several chemokines and cytokines that drive immune suppression by recruiting myeloid-derived suppressor cells [170,171,172]. Meanwhile, YAP activation in tumor-initiating cells was found to enhance the recruitment of macrophages that are essential for tumorigenesis [173]. Thus, YAP/TAZ activity in tumor cells can help protect the tumor cells from the immune system.

Although evidence suggests that, in rare cases, cancer cells can survive and grow inside the vessel, it is generally thought that exiting circulation, or extravasation, drastically enhances tumor cell survival to facilitate metastasis formation. YAP/TAZ activation can also influence this rate-limiting step of metastasis. Sharif et al. used experimental metastasis assays in both mice and zebrafish to show that YAP knockdown significantly reduces breast cancer cell extravasation and colonization [126]. Another study found that Abelson Murine Leukemia Viral Oncogene Homologs 1 and 2 (ABL1 and ABL2) promote extravasation and metastasis of lung cancer cells in a TAZ/β-catenin dependent manner [121]. 

Many aggressive cancer cells already have elevated YAP/TAZ activity before they enter circulation, but recent work suggests that entry into circulation itself may further activate YAP and TAZ. Cancer cells exposed to shear stress or disturbed flow have increased YAP or TAZ activity [174,175]. This flow-induced YAP activity promotes tumor cell motility in a TEAD-dependent manner [174]. These findings are consistent with studies in endothelial cells that show that YAP and TAZ are regulated by shear stress or flow [176,177]. Platelets, which bind cancer cells in circulation and enhance metastasis through multiple mechanisms [178], can also activate YAP in tumor cells through a RhoA-Myosin Light-Chain Phosphatase-Protein Phosphatase 1 pathway [134]. This platelet-induced YAP activation promotes anoikis resistance and is required for metastasis [134]. Thus, YAP/TAZ activation in cancer cells, either prior to entering circulation, or as a result of signals they receive in circulation, can promote tumor cell survival, protect cells from immune surveillance, and promote extravasation. Interestingly, YAP and TAZ also play important roles in endothelial cells, vascular smooth muscle cells [179], and immune cells [180,181]. This suggests that systemic treatment with YAP/TAZ inhibitors may also reduce metastasis by influencing these cell types.

#### 3.2.4. Disseminated Tumor Cell Survival, Seeding, and Metastatic Growth 

Following extravasation, a disseminated tumor cell (DTC) must be able to survive and proliferate in this foreign environment in order to grow into a metastatic tumor. Several studies implicating YAP, TAZ, or TEADs in metastasis did so using experimental metastasis assays in which tumor cells were injected directly into circulation [86,118,119,120,121,122,123,124,125,126,127,128,129,132,133,135,136,137]. Enhanced metastasis formation in these assays must be due to altered intravascular survival, extravasation, or post-extravasation survival and growth. However, it can be difficult to distinguish these processes using most in vivo metastasis assays. Nevertheless, there is evidence that suggests that YAP and TAZ can influence the survival and initial growth of DTCs after they extravasate. Three of the above studies showed that altering YAP activity not only changed the number, but also the size of the metastases that formed [124,125,133], suggesting that metastatic growth was altered. Another study showed that loss of Angiomotin promotes proliferation of cancer cells at the metastatic site in a YAP-dependent manner [119]. In addition, we found that roughly equal numbers of living control and YAP-expressing cells remained in the lungs 12 h after intravenous injection, and that, although there were slightly more YAP-expressing cells in the lungs at 24 h and 72 h post-injection, this increase was roughly equivalent to the increase in cell number observed in vitro [78]. These results suggest that in these cells YAP activation is promoting metastasis formation by enhancing survival and proliferation rather than promoting extravasation. This makes sense given that YAP and TAZ are known to drive proliferation and growth. Other studies have found that YAP or TAZ can enhance DTC survival and proliferation by influencing other cell types at the metastatic site. For example, ABL kinases promote breast cancer cell survival at the metastatic site through TAZ and Signal Transducer and Activator of Transcription 5 (STAT5)-dependent modulation of tumor cell-bone interactions [123]. YAP activation was also found to promote breast cancer metastasis to the bone by enhancing osteoclast differentiation [122]. Collectively, these studies suggest that YAP/TAZ activation enhances metastatic tumor formation by promoting post-extravasation survival and proliferation of tumor cells. This raises the possibility that YAP/TAZ inhibition could prevent the survival and outgrowth of tumor cells that have already spread.

## 4. Therapeutic Potential of Targeting YAP/TAZ-TEAD in Cancer

The experimental evidence discussed above suggests that YAP/TAZ activation, which occurs in many human cancer types, is pro-tumorigenic and pro-metastatic. Given this, there is great enthusiasm regarding YAP/TAZ-TEAD as targets for anticancer therapies and several recent reviews have discussed this [28,29,32,33,182,183,184,185]. Although YAP and TAZ activity is often elevated in cancer cells, their activity is typically low in resting tissue, and mouse models suggest that YAP and TAZ are largely dispensable in several adult organs [104,186,187]. This suggests that perhaps systemic YAP/TAZ inhibition could be used to treat cancer without causing significant adverse side effects. On the other hand, YAP and TAZ have important functions in some normal tissues where they regulate stem cell differentiation, coordinate cell proliferation, survival, and tissue repair [3,5,6]. Thus, until good therapeutic compounds that directly target YAP/TAZ-TEAD are developed and tested in humans, it is not clear how adverse the side effects will be. If directly targeting YAP, TAZ, or TEADs proves difficult or toxic, alternative approaches could target either cancer-specific pathways that promote YAP/TAZ activity in cancer cells, or the pro-tumorigenic and pro-metastatic target genes induced by YAP and TAZ. Below we discuss strategies for targeting YAP/TAZ-TEAD in metastatic cancer.

### 4.1. Directly Targeting YAP/TAZ-TEAD

The first clear example of compounds directly targeting YAP/TAZ-TEAD came from a drug screen using a YAP/TAZ-TEAD transcriptional reporter. Three porphyrin compounds (protoporphyrin ix, hematoporphyrin, and verteporfin) were found to greatly inhibit YAP/TAZ-TEAD activity [104]. One of these compounds, verteporfin, blocked YAP-TEAD interaction and suppressed YAP-mediated liver overgrowth in a transgenic mouse model [104]. Since this study, there have been numerous others that have found that verteporfin can inhibit the growth of YAP/TAZ-TEAD dependent cancer cells in vitro or in vivo [81,96,104,188,189,190,191,192,193,194,195]. These studies were promising because verteporfin is an FDA approved photodynamic therapy. However, verteporfin’s promise as a YAP/TAZ-TEAD inhibitor is outweighed by high toxicity and the accounts of YAP/TAZ-TEAD independent effects [196,197,198]. Although verteporfin prevents YAP-TEAD activity by preventing YAP-TEAD interaction [104], other evidence shows it can also activate the Hippo pathway [191], and regulate YAP and TAZ protein expression [189,190,191,195,199]. This apparent lack of specificity further diminishes its therapeutic potential. Other porphyrin compounds, including those mentioned above, also inhibit YAP/TAZ activity [104,191,197], but they are not as widely studied and additional work is needed to characterize their mechanisms of action, toxicity, and specificity. Thus, although verteporfin and other existing YAP/TAZ inhibitors are useful tools to test the impact of YAP/TAZ inhibition in pre-clinical models, they are not likely to be useful therapeutically, so new therapies need to be developed and tested. A recent study found that YAP-TEAD interaction was prevented by a cysteine-dense peptide [200], revealing another promising method of targeting the YAP/TAZ-TEAD interaction. However, this novel peptide will require additional characterization and has yet to be used in vivo. 

While directly targeting inappropriate YAP/TAZ activity holds promise, there are challenges to this approach. YAP and TAZ are transcriptional coactivators, which are classically difficult to directly target. Furthermore, most evidence suggests that it is only after YAP and TAZ translocate to the nucleus that they interact with TEADs. This means that any compound designed to prevent YAP/TAZ-TEAD interaction will need to be active in the nucleus. There is also recent evidence that the molecular flexibility of YAP influences its nuclear localization [201]. This study indicated that YAP partially unfolds to enter the nucleus, which may suggest that any bound therapeutic compound could dissociate upon nuclear entry. However, this also reveals a potential therapeutic approach. Perhaps a compound that binds YAP and stabilizes flexible regions could prevent or reduce nuclear entry. It may also be possible to target the TEADs [202]. The four TEADs share significant homology within the *N*-terminal DNA-binding domain and the *C*-terminal YAP/TAZ/VglL binding domain [185]. The structures for the DNA-binding domains have been solved [203,204,205,206,207], but there are no existing drugs known to target this region [185]. 

Other small molecules that inhibit YAP and TAZ function have also been described. However, these compounds are likely not directly targeting YAP/TAZ-TEAD, but instead are acting on upstream regulators. For example, a small molecule screen in breast cancer cells found that dasatinib, statins, and pazopanib all inhibit YAP and TAZ nuclear localization or protein stability [208]. A dipyrrin derivative called dipyrrin 19 was also found to inhibit YAP/TAZ-TEAD mediated transcriptional activity in metastatic breast cancer cells [199], and another compound called cerivastatin could prevent YAP/TAZ nuclear entry [209]. A cell-based screen of 48 chemical compounds identified dobutamine as a potent inhibitor of YAP activity [210], and subsequent studies from the same group using larger chemical compound libraries revealed several others that potently inhibit YAP [211] or TAZ [212] transcriptional activity. A small molecule, C19, can promote TAZ degradation through Hippo pathway activation, but this compound also inhibits Wnt and Transforming Growth Factor β (TGFβ) signaling [213]. Treatment with MF-438, C59, or XAV-929 reduces YAP and TAZ expression, but these are not highly specific to YAP or TAZ [214]. A combinational therapy of the histone deacetylase inhibitor, panobinostat, and the bromodomain/extra terminal protein inhibitor, I-BET151, reduces YAP protein expression through downregulation of Protein Kinase B (AKT) [215]. While these compounds that reduce YAP/TAZ nuclear localization and/or expression hold promise, it remains unclear how specific these treatments are to YAP and TAZ. Furthermore, whether they can effectively repress YAP/TAZ-mediated processes in cancer cells in vivo has not been thoroughly examined. 

### 4.2. Targeting Pathways that Activate YAP-TAZ-TEAD in Cancer

Hippo pathway mutations cannot fully explain the frequency of elevated YAP/TAZ activity observed in human cancer, which suggests that other cancer-associated pathways activate YAP or TAZ in cancer cells. Identifying these cancer-associated pathways could facilitate the development of targeted therapies to treat cancer without the potentially adverse side effects of directly targeting YAP, TAZ, or TEADs. Furthermore, identification of cancer driving pathways that promote YAP/TAZ activity may provide a diagnostic tool to help identify patients likely to respond to these targeted therapies. Numerous pathways that influence YAP/TAZ activity have been identified. Some do so by acting on the core Hippo kinase cascade, while others regulate YAP and TAZ independent of the Hippo pathway. Interestingly, FDA-approved drugs that target several of these pathways such as GPCRs, integrins, and Src already exist [216,217,218,219,220,221,222] and could be re-purposed for use in YAP/TAZ-dependent cancers. Below, we discuss several examples of regulators of YAP/TAZ that may be potential therapeutic targets for the treatment of metastatic disease.

#### 4.2.1. Src

Src is a 60-kD membrane-associated tyrosine kinase [223,224] that is activated by several protein kinases [225,226]. Src was the first proto-oncogene discovered and its role in cancer progression and metastasis is well established. Activation of Src can promote transformation, invasion, tumor growth, and metastasis [227,228,229,230,231,232,233,234,235,236,237,238,239,240], and several studies have found that Src inhibition can reduce metastasis formation in vivo [228,232,234,235,236,238]. Src levels or activity are increased in many human cancers [219,230,237,241,242,243], and Src is essential for outgrowth of disseminated tumor cells in the bone marrow [240]. 

**Figure 2:**
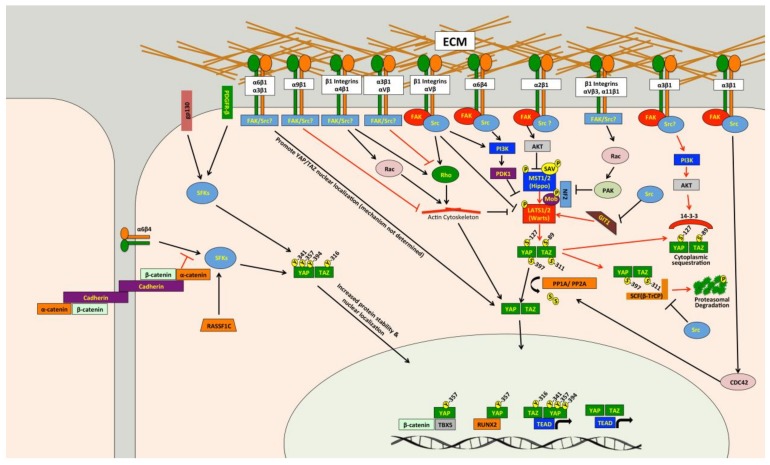
YAP/TAZ regulation by integrins and Src. Summarized are pathways that we discussed that influence YAP/TAZ activity downstream of Src or integrins. Pathways that activate YAP or TAZ are depicted as black lines, whereas inhibitory pathways are shown in red. Several of the integrin studies did not directly implicate FAK/Src signaling (indicated by FAK/Src? or Src?) but given what is known about integrin signaling cascades it is probable that they are involved.

Several studies have demonstrated that Src can promote YAP/TAZ activity through multiple independent mechanisms (Figure 2) [85,244,245,246,247,248,249,250,251,252,253,254,255,256,257,258,259,260]. Src and other Src family kinases (SFKs) can directly phosphorylate YAP [85,251,253,255,256,261] and TAZ [257] to promote their protein stability and activity. Tyrosine phosphorylation of YAP by Src or Yes was also found to promote YAP interaction with T-Box 5 (TBX5) and β-Catenin [85,256] or Runt-related Transcription Factor 2 (RUNX2) [261]. Src phosphorylates YAP on Y357 and TAZ at Y316 [257], and a recent report found that Src also phosphorylates YAP on Y341 and Y394 [251]. Src can also influence other pathways that regulate YAP and TAZ. Indeed, Src can attenuate SCF(β-TrCP) E3-ligase activity, blunting TAZ proteasomal degradation [259], and can also cause changes in the actin cytoskeleton that promote YAP/TAZ activity [254]. Several papers show that Src can repress LATS [245,248,250,252], and this appears to occur through multiple independent mechanisms. Two studies separately found that Src-mediated activation of phosphatidylinositol 4,5-bisphosphate 3-kinase (PI3K) represses LATS [248,250]. Two other studies also implicated PI3K in YAP activation mediated by integrin-Src signaling, but they did not directly implicate LATS [247,262]. Src can also repress LATS through RhoA [245]. A recent study found that Src can directly phosphorylate LATS at Y692 and Y916, which leads to decreased LATS activity [252]. In *Drosophila*, Src activity induces F-actin accumulation via the Rac-Diaphanous and Ras-mediated pathways to promote Yorkie activity [263]. Meanwhile, another study found that loss of *Drosophila* C-terminal Src kinase, a negative regulator of Src, reduces Hippo pathway repression of Yorkie [258]. In a study that we just submitted, we also identified Src as a regulator of YAP/TAZ activity [264] and, consistent with the above studies, we found that Src represses LATS in both breast cancer and melanoma cells. However, our findings suggest that in the cancer cells we tested, Src promotes YAP/TAZ activation through repression of GPCR-kinase-interacting Protein 1 (GIT1) [264], a protein known to promote LATS-mediated phosphorylation of YAP [265]. Thus, Src can influence YAP/TAZ activity through multiple pathways (Figure 2), and in a variety of cell types.

Src is often activated in cancer, suggesting that Src could be a common cause of inappropriate YAP/TAZ activity. Indeed, while some of the above studies were carried out in non-transformed cells or in *Drosophila*, several did show Src activation of YAP and/or TAZ in cancer cells [85,245,247,248,251,253,254,256,257,259]. However, it remains unclear whether all of the described Src effector pathways that regulate YAP/TAZ activity can do so in cancer. Another unanswered question is whether Src-mediated YAP/TAZ activation plays a causal role in tumor growth or metastasis. The study by Si and colleagues showed that Src-dependent YAP/TAZ activation promotes cellular transformation and enhances tumorigenicity [252]. Li and colleagues nicely demonstrated a role for Src phosphorylation of YAP in squamous cell carcinoma formation and growth [251], and Src also promotes YAP nuclear localization in epidermal papillomas and squamous cell carcinomas [247]. Another study showed that Src phosphorylation of TAZ was important for colorectal tumor formation [257]. Meanwhile, glucocorticoid receptor, which activates YAP by stimulating Src-dependent actin remodeling, is required for breast cancer growth [254]. However, none of these studies looked at the role of Src-mediated YAP/TAZ activation in metastasis. In our recently submitted study we found that Src-dependent YAP/TAZ activation is important for melanoma growth and metastasis [264], and that several metastatic melanoma and breast cancer cells require Src for YAP/TAZ activity. Collectively, these studies suggest that Src-mediated YAP/TAZ activation plays a causal role tumor growth and metastasis in some cancers, but more work is needed to determine how prevalent this pathway is in specific cancer types. If this pathway is prevalent in a significant number of cancers, then existing FDA-approved Src inhibitors [217,218,219,220,266] could be repurposed for use in these patients. This would require a means to distinguish cancers with this pathway activated from those where it is not. Another important question is whether different mechanisms of Src activation are all able to drive YAP/TAZ activity. Indeed, Src can be activated by a host of upstream pathways, and several of the studies discussed above did not determine how Src was being activated to promote YAP/TAZ activity. As discussed in detail below, integrins are clearly important drivers of Src-mediated YAP/TAZ activity [244,245,247,248,250,251,262]. Src activation downstream of either interleukin-6/gp130 [255] or platelet derived growth factor receptor (PDGFR) [253] can also promote YAP phosphorylation and activity. Figure 2 also summarizes the pathways upstream of Src regulation of YAP/TAZ. 

#### 4.2.2. Integrin-ECM Adhesion

Integrins are a family of heterodimeric transmembrane glycoproteins consisting of a α and a β subunit [267]. There are 18 different α subunits and 8 β subunits, which combine to make 24 distinct integrins [267]. Integrins interact with ECM proteins and the cytoskeleton via their extracellular and cytoplasmic domains, respectively [267]. In addition to providing physical attachment of cells to the ECM, integrins also mediate critically important signaling cascades [268]. Integrins and integrin-ECM adhesion play important roles in cancer progression and metastasis [269,270,271,272,273,274], and integrin signaling is activated in many cancers [275,276]. A fair amount of evidence showing that integrin-ECM adhesion promotes YAP/TAZ activity has emerged [244,245,247,248,250,251,262,277,278,279,280,281,282,283,284,285,286,287]. Several of these studies show that knockdown or inhibition of all β1 integrins can reduce YAP/TAZ activity in a variety of cell types [245,247,277,278,279,280]. However, these studies did not identify which of the 12 unique β1 integrins were involved, but instead suggested that integrin-mediated adhesion and spreading are generally important for YAP/TAZ activity. While it is true that there is some overlap in ligand specificity and downstream signaling among integrins, each integrin has distinct functions. Consistently, specific integrins that regulate YAP and TAZ have been identified. Indeed, the laminin-binding integrins α6β1 [281], α6β4 [248,251] and α3β1 [282,283], the collagen receptors α2β1 [284] and α11β1 [286], and the fibronectin-binding integrins αVβ3 [244,262] and α4β1 [285] can each promote YAP/TAZ activity. 

Activation of YAP and TAZ by integrins occurs through a diverse set of downstream pathways (Figure 2). Several studies show that integrins promote YAP/TAZ activation through Src [244,245,247,248,250,251,262], a key effector of integrin signaling cascades [288]. Focal Adhesion Kinase (FAK) is also likely involved in integrin-Src signaling to YAP and TAZ in many cell types [247,248,250]. Integrin-Src signaling can repress LATS, either through PI3K [247,248,250] or Rho [245], to promote YAP/TAZ activation. Meanwhile, another study found that α4β1 promotes YAP/TAZ activity through both Rho and Rac1-medaited repression of LATS [285]. Integrin-mediated Rac activation can also promote YAP activity through a different mechanism involving p21-activated kinase (PAK)-mediated phosphorylation of NF2, which reduces LATS/NF2-YAP interaction [277]. Another study found that integrins repress NF2/LATS interaction in a PAK-dependent manner [280]. Other studies also implicated PAK proteins [286,287] or Rho [278] in integrin-mediated YAP/TAZ activation, but did not test if this was LATS-dependent. Integrin α2β1 binding to collagen can also activate FAK-AKT signaling to inhibit MST1 and promote YAP activity [284]. Examples of Hippo pathway independent regulation of YAP and TAZ by integrins also exist. Indeed, α6β4 can activate Src, which can directly phosphorylate and stabilize YAP in squamous cell carcinoma cells [251]. Integrin α3β1 can promote YAP dephosphorylation and nuclear localization through a LATS-independent pathway involving FAK/Src, Cell Division Control Protein 42 Homolog (CDC42), and Protein Phosphatase-1 α subunit (PP1A) [283]. 

Given their established involvement in tumor progression and metastasis, integrins are already considered good therapeutic targets, and several FDA-approved therapies inhibit integrins [221,222]. Although these compounds performed well in preclinical models, they have enjoyed only modest success in the clinic [221,222]. Part of this failure may be due to our inability to identify cancers that are dependent upon the integrins being targeted. This is further complicated by the fact that integrin function in cancer is complex, with numerous examples of the same integrin playing seemingly contradictory roles. Consistently, some integrins also appear to have contradictory roles with respect to YAP/TAZ regulation. For example, integrin α3β1, which can promote YAP/TAZ activity in transient amplifying cells and neurons [282,283], can repress YAP/TAZ activity in keratinocytes and prostate cancer cells [289,290]. Interestingly α3β1-dependent repression of YAP occurs through a FAK/PI3K/AKT-dependent pathway yet, as described above, FAK, PI3K, and AKT have each been shown to activate YAP and TAZ in other contexts. Similarly, αVβ3, which activates YAP to promote angiogenesis [244,262], was also found to inhibit YAP in endothelial cells by repressing Rho [177]. This suggests that the same integrin can have different effects on YAP/TAZ activity that are context and cell type specific. To date, integrins α2β1, α6β1, α6β4, αVβ3 and β1 have each been shown to promote YAP/TAZ activity in cancer cells to drive proliferation, tumor formation, or tumor growth [245,248,251,280,281,284,287]. Meanwhile, integrins α9β1 and α3β1 both repress YAP in cancer cells to inhibit metastasis [144,289]. To fully exploit integrin inhibitors to treat YAP/TAZ-driven cancer, we must determine which integrins activate YAP and TAZ in a given cancer type, and we must then determine if that integrin-mediated YAP/TAZ activity influences cancer development or metastasis.

#### 4.2.3. GPCRs

GPCRs are the largest family of cell surface receptors, consisting of more than 800 members (reviewed by [216,291]). These heptahelical transmembrane proteins interact with heterotrimeric G proteins that bind to GDP when inactive. Binding to ligands or agonists promotes a conformational change that triggers the substitution of GDP with GTP binding on the Gα subunit, which leads to dissociation of the Gα-GTP subunit from Gβγ subunit. Both the Gα-GTP subunit and the Gβγ subunit are then able to stimulate biological responses [216]. There are four subtypes of Gα proteins: Gαs, Gαi/o, Gαq/11 and Gα12/13. In 2012, Yu and colleagues showed that several known GPCR ligands can regulate LATS [292]. They found that lysophosphatidic acid (LPA) and sphingosine 1-phosphate activate YAP and TAZ by inhibiting LATS through Gα12/13, whereas epinephrine and glucagon, which signal through Gαs, stimulate LATS and inhibit YAP/TAZ activity. They further showed that activation of Gαq/11 and Gα12/13 represses LATS and promotes YAP/TAZ activity; whereas activation of Gαs activates LATS and represses YAP/TAZ activity [293]. Interestingly, in a subsequent study in uveal melanoma, mutations in *GNAQ* and *GNA11* were found to promote YAP/TAZ activity [96]. This paper also showed that YAP nuclear localization is elevated in human uveal melanoma samples with these two mutations, and that verteporfin treatment can inhibit the in vivo growth of uveal melanomas with Gαq/11 mutations [96]. Another study published around that time showed that in uvea melanoma *GNAQ* mutation promotes YAP activation via a Trio-Rho/Rac signaling circuit that is independent of the core Hippo pathway [294]. Mutations in *GNAQ* and *GNA11* account for up to 83% of all uveal melanoma [205,295], which suggests that inhibition of YAP/TAZ activity, either directly or by blocking pathways downstream of GPCRs, may be an effective treatment for this cancer. There are also several other examples of GPCRs regulating YAP/TAZ activity in various cell types (reviewed in [152,292,296]). For example, in Kaposi sarcoma the viral GPCR inhibits the Hippo pathway through Gαq/11 and Gα12/13, leading to YAP/TAZ activation [297] in the cancer cells. In breast cancers, the G-Protein Coupled Estrogen Receptor was shown to inhibit the Hippo pathway and activate YAP/TAZ [298]. Several studies show that Protease-Activated Receptor 1 (PAR1) activates YAP/TAZ [299,300,301] through G12/13 and Rho-mediated inhibition of LATS [299,300]. This PAR-1-mediated YAP/TAZ activation promotes cancer stem cell-like properties, invasion, EMT, and multidrug resistance [299,301]. Importantly, PAR1 is a known driver of metastasis [302]. Thus, several GPCRs can activate YAP and TAZ to promote tumor formation and /or progression in cancer.

Although numerous other GPCRs exist, their roles in the regulation YAP and TAZ have not been directly tested. The findings from Yu et al. [292] suggest that any GPCR that signals through Gαq/11 or Gα12/13 could activate YAP and TAZ. Importantly, many studies have established roles for GPCRs in cancer progression and metastasis [303], and GPCR mutations have been described in many cancer types [304]. Altogether, this evidence suggests that targeting GPCRs, or the pathways that they activate, may be a good approach to treat cancers with elevated GPCR-mediated YAP/TAZ activity. Notably, GPCRs are direct or indirect targets of more than 50% of FDA-approved drugs [216,291], raising the possibility of repurposing some of these compounds as cancer therapies. However, as was the case for Src and integrins, this will require a more thorough understanding of which GPCRs activate YAP and TAZ to drive cancer progression and metastasis, and a way to identify cancers with these pathways activated. 

#### 4.2.4. Mechanical Cues from Tissue Microenvironment

Mechanical cues that cells receive from their tissue microenvironment have profound effects on cell behavior and can influence tissue growth, morphogenesis, and differentiation. The microenvironment of tumors is typically more rigid than that of normal tissue, which promotes cancer cell proliferation, tumor progression, and metastasis [305], and is also associated with poor prognosis [305] and reduced effectiveness of chemotherapies [306]. The first evidence that mechanical cues promote YAP/TAZ activity came in a landmark study by Dupont and colleagues [307]. They found that stiff ECM, high contractility, and increased cell spreading all promote YAP/TAZ nuclear localization and transcriptional activity. This regulation is mediated by Rho and the actin cytoskeleton, and is independent of the canonical Hippo pathway [307]. Since this study, numerous others have demonstrated that mechanical cues and cytoskeletal re-organization can influence YAP/TAZ activity, and this topic has been thoroughly discussed in recent reviews [151,308,309,310]. Collectively, these studies show that mechanical cues can influence YAP/TAZ activity through a variety of pathways, including Hippo pathway dependent and independent mechanisms. The actin cytoskeleton, actomyosin-mediated contractility, and Rho GTPases appear to be the major mediators of much of the YAP/TAZ regulation by mechanical cues, but several other proteins have also been implicated.

A stiff ECM can also promote YAP/TAZ activity in stromal cells. YAP/TAZ activation by mechanical cues promotes myofibroblast differentiation and fibrosis, and is required for the generation and maintenance of cancer-associated fibroblasts (CAFs) [246,286,311,312,313,314,315]. Enhanced YAP/TAZ activity in CAFs can drive cancer cell invasion, angiogenesis, and ECM secretion and stiffening [246,311,315]. This YAP/TAZ-mediated matrix stiffening further enhances YAP/TAZ activation in CAFs (and likely also in tumor cells), thus establishing a feed-forward loop [246]. Thus, aberrant mechanical cues in tumors can promote YAP/TAZ activity in both stromal cells and tumor cells, suggesting that preventing YAP/TAZ activation induced by abnormal mechanical cues holds promise. Directly targeting the actin cytoskeleton or actomyosin-mediated contractility are likely not good therapeutic approaches. However, compounds targeting Rho do exist and have performed well in pre-clinical models [316]. Another potential approach may be to target surface receptors that sense and respond to mechanical cues. For example, integrin-mediated adhesion is important for YAP/TAZ activation by stiff ECM [308,317] and, as described above, integrin-ECM adhesion can be targeted with existing compounds. Junctional complexes such as adherens junctions and tight junctions also play a role in sensing mechanical cues [318] and both regulate YAP and TAZ [3,31,32,33,34,35,36]. Another potential approach may be to target the underlying causes of stiff ECM in tumors. For example, Lysyl Oxidase (LOX) is an ECM crosslinking enzyme that promotes stiffer ECM to drive cancer progression and metastasis, and targeting this protein has been shown to inhibit cancer progression and metastasis [319]. This is one in a long list of enzymes and proteins that can influence the architecture and mechanical properties of the ECM, and identifying and manipulating these proteins is of great interest in the field of cancer biology. It will be interesting to see which of these proteins are able to influence cancer progression and metastasis through YAP and TAZ.

#### 4.2.5. Other YAP/TAZ Regulatory Pathways in Cancer

There are also several other pathways known to regulate YAP and TAZ that are implicated in cancer progression and metastasis. Cancer cells have well-documented alterations in metabolic pathways, and several recent reviews discuss studies demonstrating that altered metabolic pathways can promote YAP/TAZ nuclear localization [3,34,320]. Indeed, aerobic glycolysis, mevalonate synthesis, Liver Kinase B1 (LKB1), 5’ AMP-activated Protein Kinase (AMPK), salt-inducible kinases, and the Tuberous Sclerosis-mammalian Target of Rapamycin complex (TSC-mTOR) all influence YAP/TAZ activity [209,321,322,323,324,325]. Importantly, activation of YAP and TAZ by some of these metabolic pathways promotes tumorigenesis and drives cancer progression [155,322,324,326,327]. Two other pathways that have established roles in cancer development, progression, and metastasis, and have been linked to the Hippo-YAP/TAZ pathway are the TGFβ and Wnt/β-catenin pathways. Numerous papers show direct links between these pathways and YAP/TAZ regulation during both developmental and pathological processes (reviewed in [7,32,328,329,330]). There is also clear evidence that crosstalk between these pathways and YAP or TAZ is important for tumor development and cancer progression [85,331,332]. Several cancer-driving transcription factors can also regulate YAP/TAZ activity, including Twist, which can promote PAR1 to activate TAZ [301], as well as Snail, Slug, and ZEB1, which can each bind YAP and TAZ and promote their transcriptional activity [333,334,335]. Likewise, Epidermal Growth Factor Receptor (EGFR) signaling, which is altered in many cancers, regulates YAP/TAZ activity. EGFR-mediated regulation of the Hippo pathway was first described in *Drosophila* [336], and subsequent studies showed that EGFR activation inhibits the Hippo pathway to promote YAP/TAZ activity [337,338,339]. Importantly, EGFR-mediated YAP/TAZ activity can drive cancer development and progression [105,338,339,340,341]. In addition, studies also show that YAP/TAZ activation is a mechanism of resistance to EGFR inhibitors [97,342,343,344,345]. YAP can also interact directly with another EGFR family member, Erythroblastic Oncogene B4 (ERBB4), which enhances YAP transcriptional activity [92,346]. This, in turn, upregulated several EGFR family members and ligands to create a positive feedback loop that drives ovarian cancer progression [92]. Lastly, recent evidence shows that the ABL kinases, ABL1 and ABL2, enhance lung and breast cancer metastasis by promoting TAZ activity, and that knockout or inhibition of ABL kinases prevents metastasis [121,123].

The above are just a few examples of cancer-associated pathways that regulate YAP and TAZ, but many others exist and the list continues to grow. The existence of so many pathways that drive YAP/TAZ activity in cancer cells suggests that there are numerous ways we could potentially target these proteins in cancer. However, this also highlights a challenge: with so many cancer-associated pathways able to promote YAP/TAZ activity, it is unlikely that targeting just one pathway will be effective in all cancers. This means that targeting YAP/TAZ by inhibiting specific upstream pathways will require a personalized approach in which the YAP/TAZ activating pathway is identified in the patient’s cancer. A second challenge is the potential for cancers to develop resistance to these targeted therapies by activating one of the other pathways that promote YAP/TAZ activity. One reason for optimism is that several of these pathways likely converge on common signaling nodes that could be targeted. However, it is clear that more work is necessary to fully elucidate the signaling networks upstream of YAP and TAZ in cancer and to determine the frequency with which they are altered.

### 4.3. Targeting Downstream YAP/TAZ-TAZ Target Genes in Cancer Cells

Targeting the genes downstream of YAP and TAZ that drive cancer growth, progression, and metastasis is another approach that could provide therapeutic benefit and limit potential side effects, particularly if these target genes are dispensable in normal tissue. Numerous YAP/TAZ target genes are known and examples of genes that are required for YAP/TAZ-mediated tumor progression and metastasis already exist. For example, YAP drives the expression of ARHGAP29, which is required for gastric cancer metastasis [130], and YAP-mediated repression of Growth Differentiation Factor-15 promotes breast cancer metastasis [128]. YAP/TAZ-mediated expression of Neuronal Growth Regulator 1 (NERG1) and Urothelial Cancer Associated 1 Non-coding RNA (UCA1) is required for TGFβ-induced tumorigenic effects in oral squamous cell carcinoma [331]. YAP can also mediate tumorigenesis by promoting cell survival and proliferation through a Cyclooxygenase-2-EGFR signaling axis [347]. Meanwhile, YAP/TAZ-mediated expression of amphiregulin is required for the malignant behavior of breast cancer cells [115], as well as for cancer cell migration, proliferation [339,348], and transformation [349]. Upregulation of cyclin D1 and Forkhead Box Protein M1 (FOXM1) is required for YAP-driven malignant mesothelioma cell proliferation [350]. As detailed above, several YAP/TAZ target genes are required for YAP/TAZ-mediated tumor cell migration and invasion, including ARHGAP29 [130], LPAR3 [87], BMP4 [153], RHAMM [154,155], zyxin [127], and amphiregulin [339,348]. Axl is required for YAP/TAZ-dependent tumor cell invasion, proliferation, tumorigenicity, and resistance to EGFR inhibitors [91,351,352,353,354], and Axl inhibitors have performed well in pre-clinical trials [355]. Several studies establish roles for YAP/TAZ-dependent Connective Tissue Growth Factor (CTGF) and Cysteine-Rich Angiogenic Inducer 61 (CYR61) expression in cancer growth, progression, and metastasis [119,356,357,358,359,360,361]. TAZ induces lung cancer tumorigenesis by up-regulating Aldehyde Dehydrogenase 1 Family Member A1 (ALDH1A1) [93]. Many other genes regulated by YAP or TAZ in cancer cells have been described, but their importance in YAP/TAZ driven cancer has not yet been tested. 

Apart from downstream target proteins, several recent studies have revealed that YAP and TAZ also regulate the expression of microRNAs [89,282,362,363,364,365,366,367]. Bertero and colleagues found that YAP/TAZ activation promoted the expression of the miR-130/301 family, which in turn enhanced collagen deposition and ECM remodeling to further enhance YAP activity [365]. Other groups similarly found that miR-130a is regulated by YAP [89,282], and YAP-induced tumorigenesis can be reversed by inhibition of miR-130a [89]. YAP and TAZ regulate miR-25, miR-93, and miR-106b to promote non-small cell lung cancer proliferation [363], and these miRNAs are overexpressed in lung, breast, and head and neck cancers [363]. TAZ can regulate miR135b and miR224 to promote EMT and tumorigenesis in osteosarcoma [366,367]. Evidence also shows that YAP can repress miRNA processing in cancer cells [368], and that YAP and TAZ can also regulate pre-miRNA processing through Dicer [369]. These studies suggest it is likely that the list of miRNAs regulated by YAP and TAZ in cancer cells is going to grow. 

YAP and TAZ promote tumor formation, progression, and metastasis largely by regulating gene expression, which means we should be able to identify additional YAP/TAZ target genes in cancer cells that mediate these processes. However, YAP/TAZ target genes are not likely to be identical in all cancer types. Furthermore, numerous other transcription factors can interact with YAP and TAZ to influence which target genes are being regulated. This means that YAP/TAZ target genes are also likely to be context-dependent. Finally, it is likely that YAP/TAZ activation can drive the expression of numerous target genes with partially overlapping functions, which could make it difficult to identify a single protein to target. However, the fact that the above studies identified essential target genes provides some hope that this approach is viable.

## 5. Concluding Remarks

Few effective treatment options exist for patients with metastatic disease, so we desperately need to identify pathways that promote metastasis and determine how these pathways become activated in cancer cells. YAP and TAZ are overexpressed in a significant number of human cancers. Experimental evidence shows that inappropriate YAP/TAZ activation not only promotes tumor formation and growth, but also drives tumor progression and metastasis. Furthermore, YAP/TAZ activation appears to enhance multiple steps in the metastatic cascade, suggesting that YAP/TAZ inhibition could prevent, or at least slow, the spread of cancer. This makes YAP and TAZ attractive targets for cancer therapies. However, many important questions remain. For example, no studies to date have investigated whether YAP/TAZ inhibition can prevent the growth of cancer cells that have already spread. This is important because preventing the spread of cancer is not likely to help the patients who already have metastatic tumors at the time of diagnosis. A second intriguing question is whether YAP or TAZ activation is involved in the outgrowth of dormant DTCs, which is the main cause of cancer recurrence in seemingly cured patients. If so, YAP/TAZ inhibition may be effective in preventing this recurrence. DTC outgrowth and metastatic growth are both influenced significantly by the tissue microenvironment, so the fact that YAP and TAZ can drive proliferation and survival in response to a diverse set of microenvironmental cues makes it likely that they will play a role in these processes, but studies that directly test this are necessary. 

**Figure 3:**
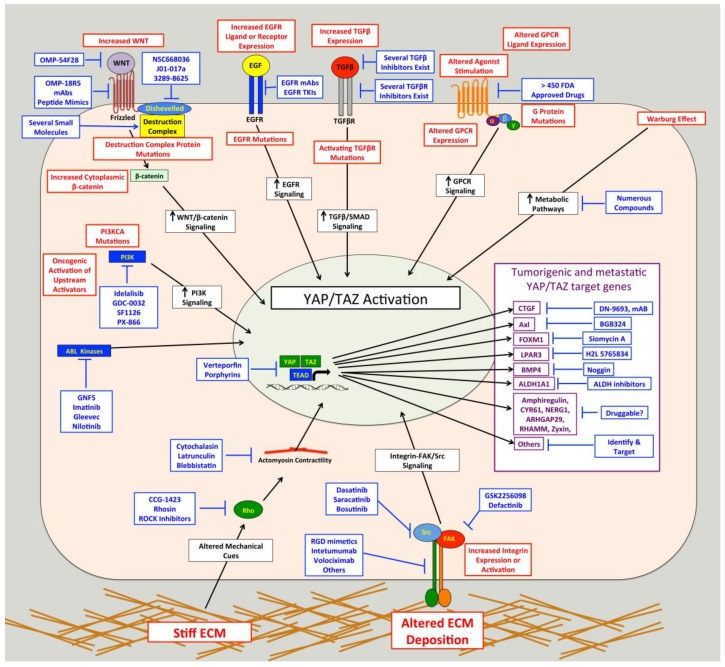
Summary of discussed cancer-relevant pathways that activate YAP and TAZ. Depicted are several pathways that activate YAP and TAZ (black boxes/text) along with an indication of how each pathway is altered in cancer (red boxes/text). YAP/TAZ target genes implicated in cancer growth and progression (purple boxes/text) are also shown. Examples of existing drugs that target these pathways or are given (blue boxes/text).

Another important question is how to actually target YAP and TAZ in cancer. Although directly targeting YAP/TAZ-TEAD seems the most obvious approach, it remains unclear how adverse the side effects of such a treatment would be. Even if they are tolerable, developing such therapies may be challenging for the reasons discussed above. Therefore, although this is an approach well worth pursuing, we also need to consider alternative strategies. As discussed above, and summarized in Figure 3, many pathways with established roles in cancer can promote YAP/TAZ activity. Importantly, there are already therapeutic compounds that inhibit several of these proteins (Figure 3). Targeting, these or other novel pathways that activate YAP and TAZ holds promise, but the downside to such an approach is that not all YAP/TAZ-driven cancers will be dependent upon the same upstream pathways. This means that targeted therapies will only be effective in a subset of YAP/TAZ-driven cancers, so a diagnostic tool to identify the cause of YAP/TAZ activation in a given tumor is essential. A systems level elucidation of the signaling networks upstream of YAP and TAZ would help identify the best pathways to target. Inhibiting pro-metastatic YAP/TAZ target genes may be another viable option (Figure 3), but this will also require a personalized approach. YAP/TAZ transcriptional signatures may hold more promise as diagnostic tools that can be employed to identify cancers with functionally active YAP or TAZ. While there is still a lot of work that needs to be done, the Hippo-YAP/TAZ pathway is a very promising target for cancer therapies, and it is encouraging how rapidly we are making progress. Close interaction and collaboration between basic scientists, clinical researchers, and pharmaceutical companies is essential to continue making progress. The annual Telluride Sciences Research Center Workshop entitled “YAP/TAZ-TEAD: at the cross roads of cancer” which inspired many of the articles in this special edition, is a great forum to promote such interaction.

## Abbreviations

YAP	Yes-Associated Protein
TAZ	Transcriptional Co-activator with PDZ-binding Motif
Mats	Mob-as-tumor Suppressor
Hpo	Hippo
Yki	Yorkie
MST1	Mammalian Sterile 20-like Kinase 1
MST2	Mammalian Sterile 20-like Kinase 2
SAV1	Salvador Homolog 1
LATS1	Large Tumor Suppressor Homolog 1
LATS2	Large Tumor Suppressor Homolog 2
MOB1A	MOB Kinase Activator 1A
MOB1B	MOB Kinase Activator 1B
TEADs	TEA Domain Family Members
FAT 1–4	FAT Atypical Cadherin 1–4
WWC1	WW and C2 Domain Containing 1
WWC2	WW and C2 Domain Containing 2
FRMD1	FERM Domain-containing Protein 1
FRMD6	FERM Domain-containing Protein 6
NF2	neurofibromin 2
Lgl1	Lethal Giant Larvae 1
Lgl2	Lethal Giant Larvae 2
ECM	extracellular matrix
GPCRs	G protein-coupled receptors
LIFR	Leukemia Inhibitory Factor Receptor
Twist	Twist-related Protein 1
ZEB1	Zinc Finger E-box-binding Homeobox 1
ABL1 and ABL2	Abelson Murine Leukemia Viral Oncogene Homolog 1 and 2
ARHGAP29	Rho GTPase Activating Protein 29
STAT5	Signal Transducer and Activator of Transcription 5
TGFβ	Transforming Growth Factor β
AKT	Protein kinase B
LIMK	LIM Domain Kinase
SCF(beta-TRCP)	Skp, Cullin, F-box(beta-transducin repeat containing)
SNAIL, SLUG	Snail Family Zinc Finger ½
LOX	Lysyl Oxidase
PD-L1	Programmed Death-Ligand 1
LPAR3	LPA Rceptor 3
TBX5	T-Box 5
RUNX2	Runt-related Transcription Factor 2
PI3K	Phosphatidylinositol 4,5-bisphosphate 3-kinase
GIT1	GPCR-kinase-interacting Protein 1
FAK	Focal Adhesion Kinase
PAK	p21-activated Kinase
CDC42	Cell Division Control Protein 42 Homolog
PP1A	Protein Phosphatase-1 α Subunit
GDP	guanosine diphosphate
GTP	guanosine triphosphate
LPA	Lysophosphatidic acid
PAR1	Protease-Activated Receptor 1
CAFs	cancer-associated fibroblasts
LKB1	Liver Kinase B1
AMPK	5’ AMP-Activated Protein Kinase
TSC	Tuberous Sclerosis
mTOR	mammalian Target of Rapamycin
EGFR	Epidermal Growth Factor Receptor
ERBB4	Erythroblastic Oncogene B4
CTGF	Connective Tissue Growth Factor
CYR61	Cysteine-rich Angiogenic Inducer 61
NERG1	Neuronal Growth Regulator 1
UCA1	Urothelial Cancer Associated 1 Non-coding RNA
BMP4	Bone Morphogenic Protein 4
RHAMM	Receptor of Hyaluronan-Mediated Motility
FOXM1	Forkhead Box Protein M1
ANKRD1	Ankyrin Repeat Domain 1
DDIT4	DNA-damage-inducible Transcript 4
TRAIL	TNF-Related Apoptosis-Inducing Ligand
ALDH1A1	Aldehyde Dehydrogenase 1 Family, Member A1
PDGFR	platelet derived growth factor receptor
TKIs	tyrosine kinase inhibitors
